# Ultra Performance Liquid Chromatography-Based Metabonomic Study of Therapeutic Effect of the Surface Layer of *Poria cocos* on Adenine-Induced Chronic Kidney Disease Provides New Insight into Anti-Fibrosis Mechanism

**DOI:** 10.1371/journal.pone.0059617

**Published:** 2013-03-26

**Authors:** Ying-Yong Zhao, Ya-Long Feng, Xu Bai, Xiao-Jie Tan, Rui-Chao Lin, Qibing Mei

**Affiliations:** 1 Key Laboratory of Resource Biology and Biotechnology in Western China, Ministry of Education, the College of Life Sciences, Northwest University, Xi’an, Shaanxi, P.R. China; 2 Solution Center, Waters Technologies (Shanghai) Ltd., Shanghai, P.R. China; 3 Research and Inspection Center of Traditional Chinese Medicine and Ethnomedicine, National Institutes for Food and Drug Control, State Food and Drug Administration, Beijing, P.R. China; ? Department of Pharmacology, School of Pharmacy, the Fourth Military Medical University, Xi’an, Shaanxi, P.R. China; Max Delbrueck Center for Molecular Medicine, Germany

## Abstract

The surface layer of *Poria cocos* (Fu-Ling-Pi, FLP) is commonly used in traditional Chinese medicine and its diuretic effect was confirmed in rat. Ultra performance liquid chromatography/quadrupole time-of-flight high-sensitivity mass spectrometry and a novel mass spectrometry^Elevated Energy^ data collection technique was employed to investigate metabonomic characteristics of chronic kidney disease (CKD) induced from adenine excess and the protective effects of FLP. Multiple metabolites are detected in the CKD and are correlated with progressive renal injury. Among these biomarkers, lysoPC(18∶0), tetracosahexaenoic acid, lysoPC(18∶2), creatinine, lysoPC (16∶0) and lysoPE(22∶0/0∶0) in the FLP-treated group were completely reversed to levels in the control group which lacked CKD. Combined with biochemistry and histopathology results, the changes in serum metabolites indicate that the perturbations of phospholipids metabolism, energy metabolism and amino acid metabolism are related to adenine-induced CKD and to the interventions of FLP on all the three metabolic pathways. FLP may regulate the metabolism of these biomarkers, especially their efficient utilization within the context of CKD. Furthermore, these biomarkers might serve as characteristics to explain the mechanisms of FLP.

## Introduction

Kidney diseases are a serious and prevalent health problem, and manifestation includes changes in renal detoxification capacity, deregulation of salt and water balance and altered endocrine functions -overall, exerting a significant impact on the patient’s short- or long-term survival. The prevalence of chronic kidney disease (CKD) continues to increase, mainly due to an increase in secondary disease as a consequence of diabetes and hypertension.

Metabonomics is a part of systems biology which refers to a holistic analytical approach to the low molecular mass organic endogenous metabolites in the tissue or bio-fluids [1]. Metabonomics provides variation of whole metabolic networks for characterizing pathological states in animals and human, as well giving diagnostic information and presenting mechanistic insight into the biochemical effects of the toxins and drugs [2]. Traditional Chinese medicines (TCM) are gaining more attention all over the world, due to their specific theory and long historical clinical practice. In the TCM research arena, this strategy has gained broad applications in many aspects, such as symptom subtyping, medicine quality control and therapeutic effect evaluation [3]–[5]. This research strategy is well coincident with the integrity and systemic feature of TCM. Mass spectrometry (MS) and nuclear magnetic resonance spectroscopy are two analytical tools commonly used in TCM metabonomics study [6]. In the MS-based metabonomics, ultra performance liquid chromatography-mass spectrometry (UPLC-MS) is considered to be suitable for large-scale untargeted metabolic profiling study due to its enhanced reproducibility of retention time [7], [8]. In 2005, Wrona *et al* introduced MS^E^ (where E represents collision energy) technique for the first time [9]. MS^E^ can provide parallel alternating scans for acquisition at either low collision energy to obtain precursor ion information, or ramping of high collision energy to obtain full-scan accurate mass fragment, precursor ion and neutral loss information [10], [11].

An adenine-induced CKD model provides valuable information of pathological mechanism for various complications in a persistent uremic state. In mammalian metabolism, excess adenine becomes a significant substrate for xanthine dehydrogenase, which oxidizes adenine to 2,8-dihydroxyadenine (DHA) via an 8-hydroxyadenine intermediate [12]. However, the very low solubility of DHA leads to precipitation in tubules of a kidney [13]. Long-term feeding of adenine to rats causes metabolic abnormalities similar to the CKD symptoms in humans. In our previous study, a metabonomic approach based on UPLC-MS was developed to characterize the metabolic profile associated with adenine-induced CKD and demonstrated that the utility of metabolic profiling combined with multivariate analysis was a powerful tool to investigate CKD pathogenesis [14]–[18].

*Poria cocos* (Schw.) Wolf (Polyporaceae) is a well-known traditional East-Asian medicinal plant that grows around the roots of pine trees in China, Japan, Korea and North America [19]. It has frequently been prescribed as one of the chief ingredients in composite prescriptions in TCM. Nearly 10% of the traditional Chinese medicinal preparations or prearations admitted to Chinese Pharmacopoeia (2010 edition) contain *Poria cocos* [20]. It is prepared from the dried sclerotia of *Poria coco*s Wolf as Fuling in China and Hoelen in Japan. The inner parts of the sclerotia of *P. cocos*, called “Fu-Ling” in Chinese, are used to treat chronic gastritis, acute gastroenteric catarrh, gastric atony, edema, nephrosis, dizziness, nausea and emesis [21].

As reported previously, the chemical constituents of *Poria cocos* mainly include triterpenes, polysaccharides and steroids [21]–[27]. However, the triterpenoid compounds is the main components of the epidermis (“Fu-Ling-Pi” in Chinese) of the sclerotia [28]–[30]. The Fu-Ling-Pi (FLP) it is believed to promote urination and to eliminate edema [20]. The diuretic effect of the ethanol and aqueous extracts of FLP has been evaluated in our recent study. The study confirmed that the not aqueous but ethanol extracts of the surface layer of *Poria cocos* presented a remarkable diuretic effect [31].

In the current study, metabonomics study based on ultra performance liquid chromatography/quadrupole time-of-flight high-sensitivity mass spectrometry (UPLC-Q-TOF/HSMS) and a novel mass spectrometry^Elevated Energy^ (MS^E^) data collection technique was applied to investigate the serum metabolite profiling of the renoprotective effect of FLP and its action mechanism. Potential biomarkers related with CKD were identified, and their metabolic pathways were also discussed. Furthermore, clinical biochemistry study were also carried out to ensure the success of the CKD model and to investigate the renoprotective effect of FLP. Therefore, metabonomics could be a promising scientific platform for therapeutic evaluation and action mechanism study of TCM.

## Experimental

### 2.1 Chemicals and Reagents

Adenine (batch No.: A8626, Purity 99.0%) and formic acid solution (ref. BCBB6918, purity 50%) were purchased from Sigma-Aldrich (St. Louis, MO, USA). Creatinine (batch No.: 100877-200901, Purity 99.8%) was obtained from the National Institutes for Food and Drug Control (Beijing, China). L-tryptophan (batch No.: TB1991-25g, Purity 99.0%) and Valine (batch No.: 1B1102-25g, Purity 99.0–101.5%) were purchased from Amresco Company (Amresco Inc., Solon, OH, USA). LC-grade methanol and acetonitrile were purchased from the Baker Company (Mallinckrodt Baker Inc., Phillipsburg, NJ, USA). Ultra high purity water was prepared using a Milli-Q water purification system (Millipore Corp., Billerica, MA, USA). Other chemicals were of analytical grade and their purity was above 99.5%.

### 2.2 Preparation of Ethanol of FLP

FLP was collected from Shaanxi Province in March 2012, and was identified by Prof. Y. Z. Wang (the College of Life Sciences, Northwest University, Xi’an, Shaanxi, P.R. China). A voucher specimen (120304) was deposited at Key Laboratory of Resource Biology and Biotechnology in Western China, Ministry of Education, Northwest University, Xi’an, Shaanxi. FLP was ground to powder (about 20 meshes) by a disintegrator, and the powder (2 kg) was extracted three times with 15 L 95% ethanol for 0.5 h by ultrasonic method. The extracts were combined together and filtrated, and then the filtrate was concentrated in vacuum using a rotary evaporator to give dried powder.

### 2.3 Animals

This study was carried out in strict accordance with the recommendations in the Guide for the Care and Use of Laboratory Animals of the State Committee of Science and Technology of the People’s Republic of China. The protocol was approved by the Committee on the Ethics of Animal Experiments of the Northwest University (Permit Number: SYXK 2010-004). All surgery was performed under uretane anesthesia, and all efforts were made to minimize suffering. All procedures and care of the rats were in accordance with the institutional guidelines for animal use in research. Male Sprague-Dawley rats were obtained from the Central Animal Breeding House of the Fourth Military Medical University (Xi’an, China). The rats were maintained at a constant humidity (ca. 60%) and temperature (ca. 23°C) with a light/dark cycle of 12 h.

### 2.4 CKD Model and Drug Administration

Male rats underwent an adaptation period of several days, during which they were fed a commercial feed. Rats weighing 200 to 220 g were divided into 3 groups (n = 8/group): group 1(control group), group 2 (CKD model group) and group 3 (FLP-treated group with CKD). Groups 2 and 3 then were then given 200 mg/kg body weight of adenine dissolved in 1% (w/v) gum acacia solution by oral gavage once everyday continuously for 2 weeks, which produced experimental renal failure in the animal for two weeks. Group 1 was similarly provided an equal volume of gum acacia solution. During the adenine gastric gavage after 3 h, Group 3 was administered FLP (60 mg/ml) by gastric irrigation. The control group and model group were only administered by oral gavage with the 1% (w/v) gum acacia solution. Body weights were recorded daily.

### 2.5 Sample Collection

After two weeks, all the rats were anesthetized with 10% urethane, and blood samples were obtained by carotid artery cannula. Blood was centrifuged at 3000 rpm for 10 min and the supernatant was collected and stored at –80°C. The rats were killed and Kidneys were collected immediately after blood was drawn and the tissues were washed with saline buffer.

### 2.6 Determination of Body Weight, Kidney Index and Blood Sample

At the end of the experiment, rats were housed individually in metabolic cages for 24 h urinary collection and body weight was measured. Kidneys were weighed to determine the organ indexes (organ index = organ weight/terminal body weight×100%).

The levels of Serum creatinine (SCr), blood urea nitrogen (BUN), cholesterol and triglyceride were determined by Olympus AU640 automatic analyzer and the levels of white blood cell count (WBC), red blood cell (RBC), hemoglobin (HGB) and hematocrit (HCT) were determined by HF-3800 Routine blood analyzer.

### 2.7 Histopathology

A portion of kidney tissue was immersed in 10% neutral buffered formaldehyde solution, the tissues were dehydrated, embedded in paraffin, cut at 5 micrometer thickness and stained with hematoxylin and eosin (H&E) for histopathological examination.

### 2.8 Preparation of Metabonomic Samples

Prior to the analysis, the serum samples were thawed at room temperature. Acetonitrile (400 µl) was added to serum (200 µl) and vortex-mixed (IKA Instruments, Guangzhou, China) vigorously for 3 min. The mixture was settled at room temperature for 10 min, and then centrifuged (eppendorf Instruments, Hamburg, Germany) at 13000 rpm for 10 min at 4°C. The supernatant (400 µl) were pipetted out and lyophilized.

### 2.9 UPLC Conditions

The UPLC analysis was performed with a Waters Acquity™ Ultra Performance LC system (Waters, USA) equipped with a Waters Xevo™ G2 QTof MS (Waters MS Technologies, Manchester, UK). Chromatographic separation was carried out at 45°C on an ACQUITY UPLC HSS (high strength silica) T3 column (2.1 mm×100 mm, 1.8 µm, UK). The mobile phase consisted of water (A) and acetonitrile (B), each containing 0.1% formic acid. The optimized UPLC elution conditions were: 0–0.5 min, 1% B; 0.5–3.5 min, 1–35% B; 3.5–7.0 min, 35–99% B; 7.0–8.0 min, 99% B and 8.0–10.0 min, 99.0–1.0% B. The flow rate was 0.45 ml/min. The autosampler was maintained at 4°C. The lyophilized serum samples were dissolved in 100 µL of distilled acetonitrile/water (4∶1). Every 2 µL sample solution was injected for each run.

### 2.10 Mass Spectrometry

Mass spectrometry was performed on a Xevo™ G2 QTof (Waters MS Technologies, Manchester, UK), a quadrupole and orthogonal acceleration time-of-flight tandem mass spectrometer. The scan range was from 50 to 1200 m/z. For both positive and negative electrospray modes, the capillary and cone voltage were set at 3.0 kV and 30 V, respectively. The desolvation gas was set to 800 L/h at a temperature of 450°C; the cone gas was set to 20 L/h and the source temperature was set to 150°C. The mass spectrometry was operated in W optics mode with 12,000 resolution using dynamic range extension. The data acquisition rate was set to 0.1 s, with a 0.1 s interscan delay. All analyses were acquired using the lockspray to ensure accuracy and reproducibility. Leucine–enkephalin was used as the lockmass at a concentration of 300 ng/mL and flow rate of 5 µL/min. Data were collected in continuum mode, the lockspray frequency was set at 10 s, and data were averaged over 10 scans. All the acquisition and analysis of data were controlled by Waters MassLynx v4.1 software.

### 2.11 Data Analysis

The mass data acquired were imported to Markerlynx XS (Waters Corporation, MA, USA) within the Masslynx software for peak detection and alignment. All of the data were normalized to the summed total ion intensity per chromatogram, and the resultant data matrices were introduced to the EZinfo 2.0 software for principal component analysis (PCA) and partial least squares-discriminate analysis (PLS-DA) analyses. Metabolite peaks were assigned by MS^E^ analysis or interpreted with available biochemical databases, such as HMDB (http://www.hmdb.ca/), Chemspider (http://www.chemspider.com) and KEGG (http://www.kegg.com/). Other statistical analyses were used by one-way analysis of variance (ANOVA) and independent sample *t*-test. They were performed with SPSS 11.0 (SPSS Inc., Chicago, IL, USA). Significant differences were considered significant when test *P* values were less than 0.05.

## Results and Discussion

### 3.1 Basic Physical Parameters

**Figure 1:**
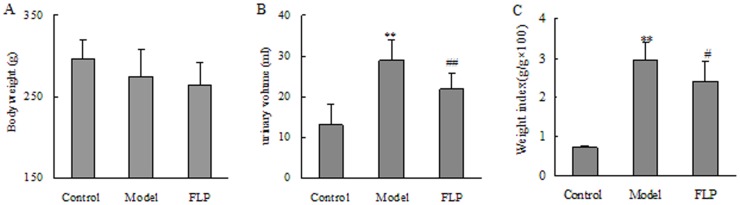
Physical parameter comparisons among the studied groups. (A) Body weight; (B) urinary volume and (C) kidney weight index. The data were expressed as mean ± SD. *P<0.05, **P<0.01 compared to the control groups and ^#^ *P*<0.05, ^##^ *P*<0.01 compared to the CKD groups.

Figure 1 shows the parameters of body weight, urinary volume and kidney weight index among the studied groups. Body weight was decreased in the CKD group compared with that in the control group, but did not arrive at statistical significance. Similarly, compared with the CKD group, body weight was also slightly decreased in the FLP-treated group. Because FLP is used as a diuretic in clinic, decrease of weight body can be caused by FLP’s diuretic effect. Urinary volume was markedly increased in the CKD group compared with that in the control group and arrived at statistical significance (*P*<0.01), but decrease of urinary volume was revealed in the FLP-treated group.

### 3.2 Biochemical Parameters

**Figure 2:**
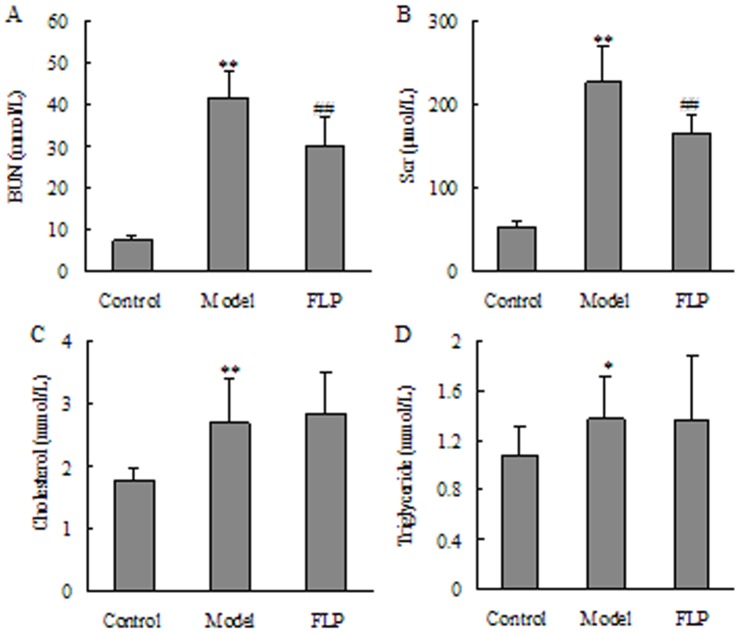
Basic serum biochemical parameter comparisons among the studied groups. (A) BUN; (B) Scr; (C) cholesterol; (D) triglyceride. The data were expressed as mean ± SD. *P<0.05, **P<0.01 compared to the control groups and ^#^ *P*<0.05, ^##^ *P*<0.01 compared to the CKD groups.

After day 14, blood samples were collected and BUN, Scr, cholesterol and triglyceride were determined. The results are given in Figure 2. BUN, Scr, cholesterol and triglyceride were all in higher concentrations in CKD group than in control group (*P*<0.01). These results demonstrate that the rat model exhibited typical pathologic features associated with CKD. Levels of BUN and Scr were significantly intervened by FLP (*P*<0.01).

**Figure 3:**
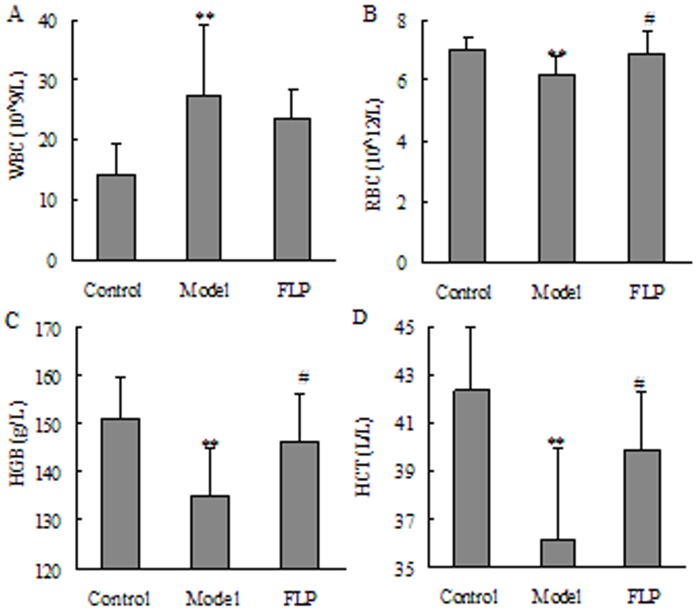
Basic blood routine parameters of the studied groups. (A) White blood cell count; (B) red blood cell; (C) hemoglobin and (D) hematocrit. The data were expressed as mean ± SD. *P<0.05, **P<0.01 compared to the control groups and ^#^ *P*<0.05, ^##^ *P*<0.01 compared to the CKD groups.

The results of blood routine are given in Figure 3. A remarkable increase in WBC and a remarkable decrease in RBC, HGB and HCT were revealed in the blood parameters of the CKD group compared with the control group (*P*<0.01). These blood parameters showed that adenine can cause anemia symptom. FLP could increase RBC, HGB and HCT to the same extent although they could not increase RBC, HGB and HCT to the normal levels. These results demonstrate that the CKD was being prevented and alleviated, exhibiting a recovery via similarity to the healthy control group after taking FLP.

### 3.3 Histological Results

**Figure 4:**
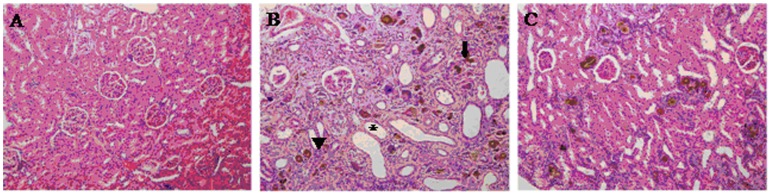
Histologic findings by HE-staining of transverse kidney sections. (A) normal control rats, (B) adenine-induced CKD rats and (C) FLP-treated group with CKD rats. Large dilated tubule (asterisk); lymphocytic infiltrate (arrowheads); 2,8-dihydroxyadenine crystal (arrows). There was formation of foreign body granuloma in the renal tubules and interstitium and a marked fibrosis leading, in some extreme cases, to a contracted kidney. These results demonstrate that the rat model exhibited the typical pathological features associated with CKD.

Figure 4 illustrates the histological findings obtained through the HE staining of transverse kidney sections from the adenine-induced rats. There was formation of foreign body granuloma in the renal tubules and interstitium and a marked fibrosis leading, in some extreme cases, to a contracted kidney (Figure 4B). These results demonstrate that the rat model exhibited the typical pathological features associated with CKD. In contrast, these pathological abnormalities were gradually ameliorated in the FLP-treated group (Figure 4C).

### 3.4 Method Development and Validation

**Figure 5:**
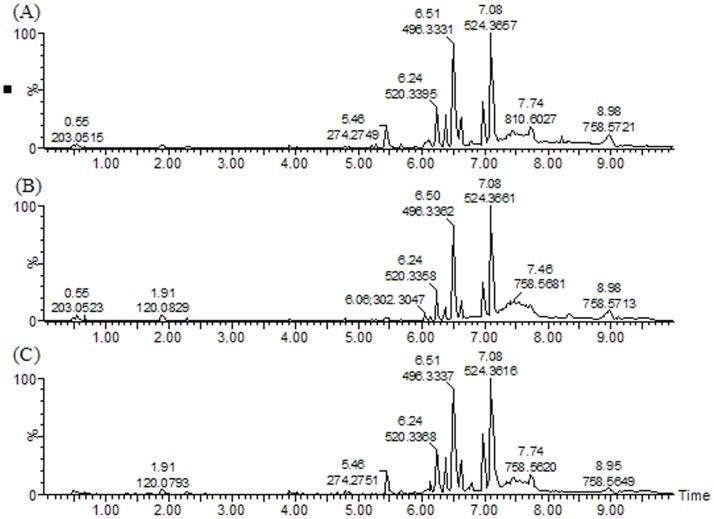
The UPLC-MS base peak intensity (BPI) chromatogram. (A) control group, (B) CKD group and (C) FLP-treated group.

High reproducibility is crucial for any analytical protocols, especially for metabonomics study which requires handling many samples. The precision and repeatability of the UPLC-MS method were validated by the reduplicate analysis of six injections of the same quality control samples and six parallel samples prepared using the same preparation method, respectively. The relative standard deviations (RSD) of retention time and peak area are below 0.45% and 3.4%, respectively. The resulting data showed that the precision and repeatability of the proposed method were satisfactory for metabonomic analysis. Metabolic profiling of serum samples was acquired using UPLC Q-TOF/MS system in the positive ion mode. The base peak intensity (BPI) chromatograms of serum samples from healthy control group, CKD group and FLP-treated group are shown in Figure 5.

### 3.5 Biomarker Elucidation

**Figure 6:**
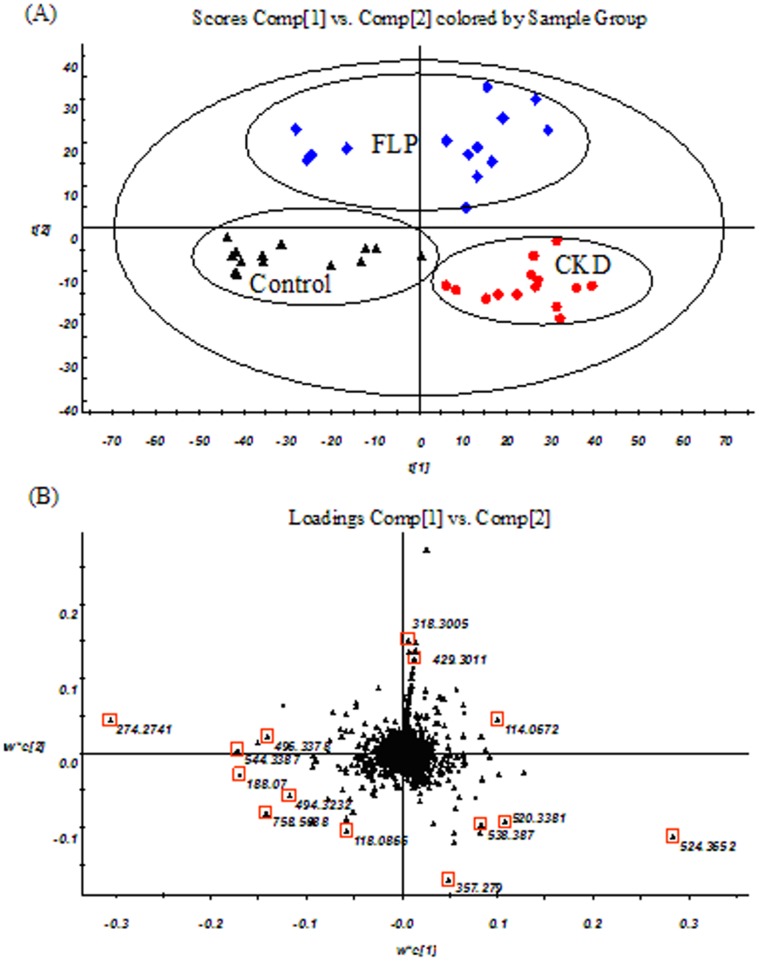
PLS-DA scores plots (A) and Loading plots (B) derived from UPLC-MS data of serum samples. (▴) control group, (•) CKD group and (♦) FLP-treated group. The variables marked (□) are the metabolites selected as potential biomarkers.

To determine whether the FLP was possibly to influence metabolic pattern of adenine-induced CKD rats and to find the metabolites with a significant concentration change (i.e. potential biomarkers), PLS-DA was carried out on the UPLC-MS data of serum samples. A PLS-DA model was built by 2112 variables. Figure 6 shows the score plot and the loading plot in the positive ion mode. The score plot shows that control, CKD and FLP-treated groups are classified clearly, which might suggest FLP has a protect effect on adenine-induced CKD model.

In the score plot, scattered points of various samples were classified into three groups, which suggested that proper CKD-related patterns could be revealed by the proposed PLS-DA model and serum metabolic pattern significantly changed after the treatment of FLP. The loading plot displayed 20 potential CKD-related metabolites according to their VIP (Variable Importance in the Projection) values. To identify these metabolites, we first searched candidates from the databases of HMDB (http://www.hmdb.ca/), Chemspider (http://www.chemspider.com/) and KEGG (http://www.kegg.com/) by masses and MS^E^ data, and due to the possible fragment mechanisms, items without the given mass fragment information were removed from the candidate list and only the most probable items survived. MassLynx i-FIT algorithm is used to screen suggested elemental compositions by the likelihood that the isotopic pattern of the elemental composition matches a cluster of peaks in the spectrum, increasing confidence in identified compounds and simplifying results. The lower the i-FIT value, the better the fit. By comparing the retention times and mass spectra to the authentic chemicals, a part of the CKD-related metabolites were structurally confirmed.

**Figure 7:**
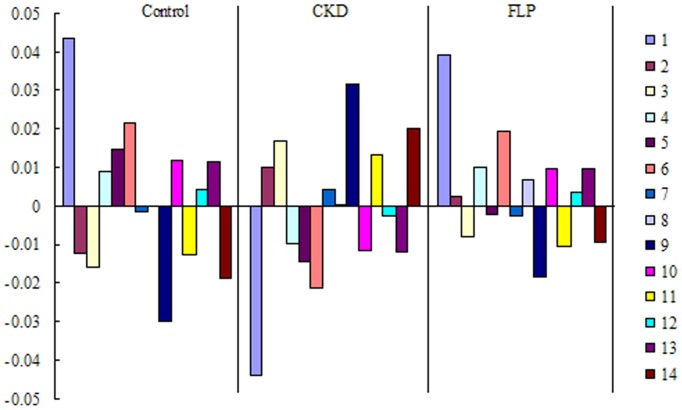
Correlation coefficient analysis between groups with corresponding markers in different groups. Variables are presented in control, CKD and FLP groups. Values of correlations are shown in the vertical axis (upper for positive correlations and low for negative correlations) and corresponding markers represented to the right of the bars. Numbers are consistent with Table 1.

Table 1 shows 14 compounds were tentatively identified on the basis of MS^E^ fragmentation data and i-FIT values, including phospholipid, amino acids and other compounds. The metabolites shown in Table 1 were ranked in the order of their VIP values, such that metabolites listed in the front were more important than those in the rear. Most of these metabolites display CKD-related changes and the CKD-related changes are partly displayed in Figure 7. Many of these identified metabolites have also been reported in other CKD studies, such as phytosphingosine [16], PC(16∶0/18∶2) [16], tryptophan [16], lysoPC(16∶1) [14], creatinine[14] and lysoPC (16∶0) [14], while in the current study, the approach of metabonomics was employed and more CKD-related metabolites were discovered.

**Table 1:** Identification of significantly differential endogenous metabolites in the rat kidney.

No	*t*_R_	m/z	Scan mode	Qusi-molecular ion	Metabolite	Trend^a^	Trend^b^	Related pathway
1	5.47	274.2741	+	[M+NH_4_]^+^	Palmitic acid	↓***	↑***	Fatty acid metabolism
		255.2317	−	[M−H]^−^				
2	7.09	524.3652	+	[M+H]^+^	LysoPC(18∶0)	↑***	↓***	Phospholipid metabolism
		522.3565	−	[M−H]^−^				
3	5.27	357.2790	+	[M+H]^+^	Tetracosahexaenoic acid	↑***	↓***	Fatty acid metabolism
		355.2641	−	[M−H]^−^				
4	5.48	318.3005	+	[M+H]^+^	Phytosphingosine	↓	↑**	Phospholipid metabolism
		316.2851	−	[M−H]^−^				
5	7.70	758.5688	+	[M+H]^+^	PC(16∶0/18∶2)	↓**	↑	Phospholipid metabolism
		756.5547	−	[M−H]^−^				
6	2.30	188.0700	+	[M+H−NH_3_]^+^	Tryptophan	↓***	↑**	Phenylalanine, tyrosine, tryptophan bioaynthsis
		203.0795	−	[M−H]^−^				
7	6.22	544.3387	+	[M+H]^+^	LysoPC(20∶4)	↓***	↑**	Phospholipid metabolism
		542.3250	−	[M−H]^−^				
8	6.31	427.2848	+	[M+H]^+^	Prostaglandin PGE_2_ glyceryl ester	↑	↑***	arachidonic acid metabolism
		425.2544	−	[M−H]^−^				
9	6.22	520.3381	+	[M+H]^+^	LysoPC(18∶2)	↑***	↓***	Phospholipid metabolism
		518.3251	−	[M−H]^−^				
10	6.04	494.3232	+	[M+H]^+^	LysoPC(16∶1)	↓***	↑	Phospholipid metabolism
		492.3092	−	[M−H]^−^				
11	0.58	114.0672	+	[M+H]^+^	Creatinine	↑***	↓***	Arginie and proline metabolism
		112.0482	−	[M−H]^−^				
12	6.36	496.3378	+	[M+H]^+^	LysoPC (16∶0)	↓***	↑**	Lipid metabolism
		494.3278	−	[M−H]^−^				
13	0.58	118.0866	+	[M+H]^+^	Valine	↓**	↓**	ABC transporters
		116.0717	−	[M−H]^−^				
14	7.37	538.3870	+	[M+H]^+^	LysoPE(22∶0/0∶0)	↑**	↓**	Lipid metabolism
		536.3768	−	[M−H]^−^				

a Change trend of CRF rats vs control rats.

b Change trend of FLP rats vs CRF rats.

The levels of potential biomarkers were labeled with (↓) down-regulated and (↑) up-regulated (*P<0.05; **P<0.01; ***P<0.001).

### 3.6 Biochemical Interpretation

**Figure 8:**
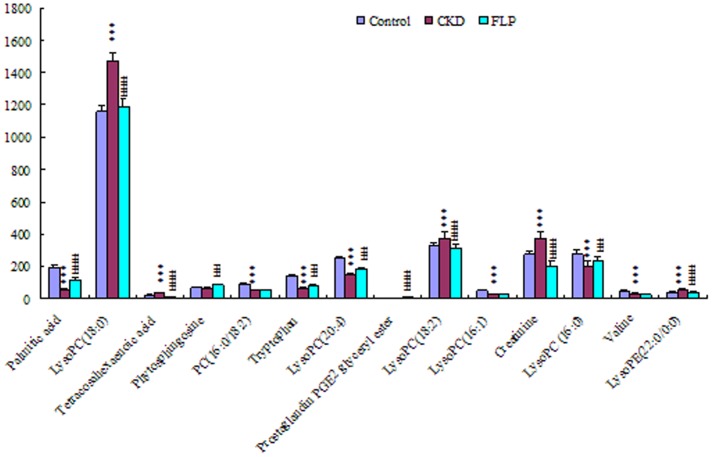
Differential expression levels (mean) of biomarkers in different groups. The “asterisk” indicated the statistical significance of the biomarkers changes by Student’s t-test: *P<0.05, **P<0.01, ***P<0.001 significant difference compared with control group; ^#^ *P*<0.01, ^##^ *P*<0.01, ^###^ *P*<0.001 significant difference compared with CKD groups.

In order to more clearly characterize treatment CKD effects of FLP, a correlation coefficient analysis was applied to investigate the connections between biomarkers and corresponding groups (Figure 7). Variables situated upper are positively correlated to group and those situated opposite are negatively correlated to the group. The markers 2, 3, 9, 11, 14 such as lysoPC(18∶0), tetracosahexaenoic acid, lysoPC(18∶2), creatinine, lysoPE(22∶0/0∶0) have negative correlation with the control group; others have positive correlation with the control group, indicating normal kidney function. Correlations between markers 1, 5, 6, 10, 13 with the control group while being relatively high when compared to the other variables, therefore the change in palmitic acid, PC(16∶0/18∶2), tryptophan, lysoPC(16∶1), valine has the strongest association with normal kidney function and are enough to suggest that these markers can principally represent the CRF model in this study. The markers 2, 3, 7, 9, 11, 14 have positive correlation with the model group; others have negative correlation with the model group, shows the overall metabolic profile of adenine caused a significant CKD. The markers 1, 2, 4, 6, 8, 10, 12, 13 have positive correlation with the FLP group; others have negative correlation with the FLP group, consistenting with the control group. The metabolism pathway of each biomarker was shown in Table 1 by searching the KEGG database. The 14 biomarkers were mainly distributed in related pathways of phospholipids metabolism, fatty acids metabolism and amino acids metabolism. In the FLP-treated rats, lysoPC(18∶0), tetracosahexaenoic acid, prostaglandin PGE_2_ glyceryl ester, lysoPC(18∶2), creatinine and lysoPE(22∶0/0∶0) were up-regulated, but palmitic acid, phytosphingosine, PC(16∶0/18∶2), tryptophan, lysoPC(20∶4), lysoPC(16∶1), lysoPC (16∶0) and valine were down-regulated compared with control rats. To evaluate protective effects of FLP, the intensity level of 14 biomarkers in the different groups was also analyzed (Figure 8). Except for PC(16∶0/18∶2), prostaglandin PGE_2_ glyceryl ester, lysoPC(16∶1) and valine, mean level of the key biomarkers was reversed to control at different degrees after oral administration (Figure 8). Among these biomarkers, lysoPC(18∶0), tetracosahexaenoic acid, lysoPC(18∶2), creatinine, lysoPC (16∶0) and lysoPE(22∶0/0∶0) in the FLP-treated group were completed reversed to levels in the control group which lacked CKD. Thus, FLP may regulate the metabolism of these biomarkers, especially their efficient utilization within the context of CKD. Furthermore, these biomarkers might serve as characteristics to explain the mechanisms of FLP. Interestingly, the most important CKD-related metabolites were phospholipid and in total there were eight phospholipids, which accounts for 60% of all the identified biomarkers. Hereby, the pathway of phospholipids metabolism was discussed in the process of renal injury.

Lysophosphatidylcholines are a class of compounds that have a constant polar head, and fatty acyls of different chain lengths, position, degrees of saturation, and double bond location in plasma. Lysophosphatidylcholine level can be a clinical diagnostic indicator that reveals pathophysiological changes. Lysophosphatidylcholines are products or metabolites of phosphatidylcholines, which are structural components of animal cell membranes. The importance of plasma phospholipid abnormalities in renal damage is well recognized [32]–[34], but little attention has been paid to the study of some plasma phospholipid fractions, including lysophosphatidylcholine, which might be expected to be important factors in the pathogenesis of the renal damage. This study indicated that up-regulated lysoPC(18∶0), lysoPC(18∶2) and lysoPE(22∶0/0∶0) and down-regulated lysoPC (16∶0) were obviously observed in adenine-induced CKD group (Figure 8 and Table 1), and the reason is not completely clear. It is reported that oxidative stress is related to renal damage [35]–[37]. Clearly, patients with CKD undergo high oxidative stress because of decreasing antioxidant defenses and increasing prooxidant factors. Several pathophysiologic explanations were put forward. Some attribute the high oxidative stress to malnutrition and hypoalbuminemia, and others propose an association of comorbid factors such as advanced age, diabetes, and inflammatory and infectious phenomena [38], [39]. When oxidative stress occurred, the generation of free radical can activate the phospholipase A2, which could hydrolyse phosphatidylcholine to produce lysophosphatidylcholine. This fact may explain the increasing trend of lysophosphatidylcholine in adenine-induced CKD group. Recent literature indicated that lysophosphatidylcholine, by activating protein kinase C signaling pathways, stimulates epidermal growth factor receptor transactivation and down-stream mitogen-activated protein kinase signaling resulting in mesangial hypercellularity, which is a characteristic feature of diverse renal diseases [40].

Creatinine is another potential biomarker for the separation of CKD rats and FLP-treated group. Increase of creatinine was observed in serum metabolite profiles of CKD group compared with control group and decrease of creatinine was revealed in the serum metabolite profiles of FLP-treated group. Creatinine is an important biomarker to evaluate renal function. This finding corresponded to the results measured with biochemical method in clinical practice. It indicated that UPLC–MS technique and PLS-DA pattern classification are credible. Creatinine is a nonenzymatic breakdown product of creatine and phosphocreatine, and the creatine-phosphocreatine system is crucial for cellular energy transportation. It is reported that animal models with adenine-induced CRF is associated with progressive renal disturbances [16].

### Conclusions

A metabonomics method based on UPLC-MS has been developed to study the effects of FLP on adenine-induced CKD rats. Multivariate statistical analysis shows a clear separation among control group, CKD group and FLP-treated group. Some potential biomarkers like lysoPC(18∶0), tetracosahexaenoic acid, lysoPC(18∶2), creatinine, lysoPC (16∶0) and lysoPE(22∶0/0∶0) have been identified. Combined with biochemistry and histopathology results, the changes in serum metabolites indicated pharmacological effects of FLP are related to phospholipids metabolism, energy metabolism and amino acids metabolism. The work shows that the metabonomics method is a valuable tool in drug mechanism research.
